# Measuring cognitive impairment and monitoring cognitive decline in Huntington’s disease: a comparison of assessment instruments

**DOI:** 10.1007/s00415-023-11804-0

**Published:** 2023-07-18

**Authors:** Andrea Horta-Barba, Saul Martinez-Horta, Jesús Pérez-Pérez, Arnau Puig-Davi, Natascia de Lucia, Giuseppe de Michele, Elena Salvatore, Stefanie Kehrer, Josef Priller, Simone Migliore, Ferdinando Squitieri, Anna Castaldo, Caterina Mariotti, Veronica Mañanes, Jose Luis Lopez-Sendon, Noelia Rodriguez, Asunción Martinez-Descals, Filipa Júlio, Cristina Januário, Marianna Delussi, Marina de Tommaso, Sandra Noguera, Jesús Ruiz-Idiago, Emilia J. Sitek, Renata Wallner, Angela Nuzzi, Javier Pagonabarraga, Jaime Kulisevsky, the Cognitive Phenotype Working Group of the European Huntington’s Disease Network

**Affiliations:** 1 Department of Medicine, Autonomous University of Barcelona (UAB), Bellaterra, Spain; 2 Movement Disorders Unit, Neurology Department, Hospital de La Santa Creu I Sant Pau, Barcelona, Spain; 3 Sant Pau Biomedical Research Institute (IIB Sant Pau), Barcelona, Spain; 4 Centro de Investigación en Red-Enfermedades Neurodegenerativas (CIBERNED), Madrid, Spain; 5 European Huntington’s Disease Network (EHDN), Ulm, Germany; 6 University of Naples “Federico II”, Naples, Italy; 7 Department of Neuropsychiatry, Charité-Universitätsmedizin, Berlin, Germany; 8 Huntington and Rare Diseases Unit, Fondazione IRCCS Casa Sollievo Della Sofferenza Research Hospital, San Giovanni Rotondo, Italy; 9 Fondazione IRCCS Istituto Neurologico Carlo Besta, Milan, Italy; 10 Department of Neurology, Hospital Universitario Ramon Y Cajal, Madrid, Spain; 11 Department of Neurology, Fundación Jimenez Diaz, Madrid, Spain; 12 Coimbra Institute for Biomedical Imaging and Translational Research-CIBIT, University of Coimbra, Coimbra, Portugal; 13 Neurology Department, Coimbra University Hospital, Coimbra, Portugal; 14 Applied Neurophysiology and Pain Unit, Apulian Center for Huntington’s Disease SMBNOS Department, “Aldo Moro” University, Bari, Italy; 15 Hospital Mare de Deu de La Mercè, Barcelona, Spain; 16 Department of Neurological and Psychiatric Nursing, Faculty of Health Science Medical, University of Gdansk, Gdańsk, Poland; 17 Department of Neurology, St. Adalbert Hospital, Copernicus, Gdańsk, Poland; 18 Department of Psychiatry, Medical University of Wroclaw, Wroclaw, Poland

**Keywords:** Huntington’s disease, Cognition, Neuropsychology, Disease progression, Mild cognitive impairment, Dementia

## Abstract

**Background:**

Progressive cognitive decline is an inevitable feature of Huntington’s disease (HD) but specific criteria and instruments are still insufficiently developed to reliably classify patients into categories of cognitive severity and to monitor the progression of cognitive impairment.

**Methods:**

We collected data from a cohort of 180 positive gene-carriers: 33 with premanifest HD and 147 with manifest HD. Using a specifically developed gold-standard for cognitive status we classified participants into those with normal cognition, those with mild cognitive impairment, and those with dementia. We administered the Parkinson’s Disease-Cognitive Rating Scale (PD-CRS), the MMSE and the UHDRS cogscore at baseline, and at 6-month and 12-month follow-up visits. Cutoff scores discriminating between the three cognitive categories were calculated for each instrument. For each cognitive group and instrument we addressed cognitive progression, sensitivity to change, and the minimally clinical important difference corresponding to conversion from one category to another.

**Results:**

The PD-CRS cutoff scores for MCI and dementia showed excellent sensitivity and specificity ratios that were not achieved with the other instruments. Throughout follow-up, in all cognitive groups, PD-CRS captured the rate of conversion from one cognitive category to another and also the different patterns in terms of cognitive trajectories.

**Conclusion:**

The PD-CRS is a valid and reliable instrument to capture MCI and dementia syndromes in HD. It captures the different trajectories of cognitive progression as a function of cognitive status and shows sensitivity to change in MCI and dementia.

## Introduction

Huntington disease (HD) is a neurodegenerative disorder caused by a CAG repeat expansion in the HTT gene. It is characterized by the progressive development of motor, cognitive and behavioral alterations. Although clinical diagnosis is based on the presence of unequivocal motor abnormalities, almost all individuals with HD develop a gradual process of cognitive deterioration that runs from mild cognitive impairment, with minimal impact on functional independence, to frank dementia with a dramatic impact on functionality [1–5]. Moreover, relatively subtle cognitive and behavioural changes often precede the motor-based diagnosis of HD by several years [6–8]. However, given the lack of HD-specific approaches to the diagnosis of the various cognitive syndromes occurring in HD [9], the prevalence of the different categories of cognitive impairment at different stages of the disease remains unclear. Besides, the clinimetric properties of the cognitive instruments in use are presently underdeveloped [10].

While deficits in frontal-executive function, attention, and processing speed have been conceptualized [11, 12] as the most characteristic and representative cognitive features of the disease [13, 14], cognitive impairment in HD appears to be more complex, and several cognitive domains may be affected along the trajectory of neurodegeneration [15–17]. Large multicentric studies, such as the Enroll-HD Study [18], have focused on identifying reliable measures that offer sufficient sensitivity to track disease progression over time. Several cognitive measures with a strong dependence on frontal-related processes, but not devoid of a motor component (i.e.: the Symbol Digit Modality Test (SDMT) or the Stroop Word Reading Test (SWRT), have demonstrated a strong sensitivity to change [13, 19, 20]. Such measures have become essential components when building composite scores to monitor HD progression [21, 22]. Still, compared with measures for other neurological diseases, instruments currently available are insufficient to classify the degree of CI (i.e.: MCI, dementia) in the HD population. What´s more, this deficiency cannot be solved on the basis of addressing a single cognitive process or domain, such as when using the SDMT or the SWRT score. Currently, the only diagnostic approach available is that proposed by the DSM-5 and the use of cutoffs established in other diseases [23–26]. At the present time, therefore, it is difficult to select HD subjects for inclusion in studies of cognitive modifiers and to rate changes in cognition in these patients.

To further our knowledge regarding the utility of the currently instruments we took advantage of the collaborative effort of an international task force and designed a prospective multicentre study in which we administered a set of cognitive tests that addressed global cognitive function. Using a reliable gold-standard for cognitive status, we determined the prevalence of various categories of cognitive impairment in different HD groups. We then addressed and compared the discriminative properties of the UHDRS cog-score and two other global cognitive instruments that have also been used in HD (the MMSE and the PD-CRS). We determined the sensitivity to change of these assessments in the overall sample and in each cognitive group.

## Participants and methods

### Participants

This multicentre international HD study was coordinated by the Cognitive Phenotype Working Group (CPWG) of the European Huntington’s Disease Network (EHDN). Subjects were recruited from 10 centers in Spain, Italy, Germany, Portugal and Poland, and all were participants in the Enroll-HD study [18]. All participants were adults with no neurological disorder other than HD and free of concomitant illnesses such as history of head injury, drug/alcohol abuse, severe language difficulties, and major psychiatric illness potentially interfering with their performance.

We included 180 gene mutation carriers (CAG ≥ 39). Participants were classified as premanifest HD (PreHD) if they had a Unified Huntington’s Disease Rating Scale (UHDRS) total motor score (TMS) below 5, a diagnostic confidence level (DCL) < 3 and a Total Functional Capacity (TFC) of 13. They were classified as manifest HD (HD) if they had a UHDRS-TMS > 4 and a diagnostic confidence level (DCL) = 4, which means that motor abnormalities are unequivocal signs of HD with ≥ 99% confidence. We calculated the disease burden score assumed as a measure of lifelong exposure to mutant HTT using the formula DBS = [(CAG − 35.5) × age] [27].

### Assessments

We recorded clinical and sociodemographic data regarding age, gender, education, and CAG repeat length. The severity of motor symptoms was rated using the Unified Huntington´s Disease Rating Scale Total Motor Score (UHDRS-TMS). The TFC and the UHDRS Independence Scale (IS) were administered to assess patients´ functionality [28]. Disease stage was determined based on Shoulson and Fahn criteria [29]. Therefore, patients were classified as Stage I when TFC > 10, Stage II when TFC was between 6 and 10, and Stage III when TFC < 6. Patients in stage IV and V were excluded.

To meet the minimum standards to deal with the lack of a validated gold standard for global cognitive status in HD, and in absence of specific and well-defined criteria, we developed a two-step approach similar to those previously used [4, 23]. The first step was the administration of the Clinical Dementia Rating Scale (CDR) [30] and the Functional Independence Score (FIS). The CDR is a global rating scale which assesses performance in memory, orientation, judgment and problem solving, community affairs, home and hobbies, and personal care. It is based on a scale from 0 to 3, where 0 = no cognitive impairment (CDR = 0), 0.5 = mild cognitive impairment (CDR =), and ≥ 1 = moderate to severe dementia (CDR). In a second step, a rater blinded to the CDR and FIS score collected the clinician´s impression of the patient’s cognitive status based on a Likert-like scale, where 0 = absence of significant cognitive impairment, 1 = minor or mild cognitive impairment, and 2 = major cognitive impairment or dementia. Based on this two-step procedure, patients were classified into a given cognitive category when there was an agreement of 100% between the two raters. Accordingly, patients were categorised as cognitively normal (HD-NC) when they had a CDR = 0, a FIS score > 80%, and a second rater impression = 0. Cognitive impairment (HD-MCI) was considered mild if a CDR = 0.5, a FIS score > 80% and a second rater impression was 1. Dementia (HD-Dem) was categorized as a CDR > 0.5, a FIS score < 80%, and a second rater’s impression of 2 [4, 23, 31].

### Neuropsychological assessment

Global cognitive status was assessed using the Mini-Mental State Examination (MMSE) [32] and the Parkinson’s Disease-Cognitive Rating Scale (PD-CRS) [33] as screening measures. The MMSE is part of the Enroll-HD protocol and the PD-CRS has recently shown to be a reliable instrument to assess global cognition in HD [23]. The MMSE covers orientation to time and place, attention-concentration, short-term memory, language skills, visuo-constructional abilities and ability to understand and follow instructions. The total MMSE score provides a general measure of global cognition and various cut-off scores are used to classify patients into cognitive categories ranging from mild cognitive impairment to severe dementia. The PD-CRS is composed of 9 subtests that assess immediate verbal memory, confrontation naming, sustained attention, verbal working memory, unprompted drawing of a clock, copy of a clock, delayed free recall, alternating verbal fluency and action verbal fluency. The PD-CRS provides a total score that both in HD and in Parkinson’s disease demonstrated to be psychometrically reliable in terms of differentiating patients into cognitive categories [33].

Cognition was also assessed using the set of cognitive tests comprising the UHDRS cognitive section or “cogscore”. Accordingly, we administered the Stroop color-naming, the word-reading and interference tests, the phonetic verbal fluency test with letters F, A and S (FAS), and the Symbol Digit Modalities Test (SDMT). There is no standard regarding the meaning of the total UHDRS cogscore in terms of classification of the severity of cognitive impairment based in this measure. Following randomization, the tests were administered at baseline visit and at two consecutive follow-up visits at 6 and at 12 months.

The study was approved by the local ethics committee at Hospital de la Santa Creu i Sant Pau, Barcelona, and reviewed by the institutional review board at each participating institution. The study was conducted in accordance with the 1964 Helsinki Declaration and its later amendments. All procedures were performed after completion of the written informed consent signed by each participant. The data used in this study are available under request.

### Statistical analysis

Data are expressed as means ± standard deviation (SD) for the continuous variables and as mean range for the ordinal variables. Differences between groups were analyzed using independent two-tailed *t* tests and analyses of variance (ANOVA) for continuous variables, the Mann–Whitney test for ordinal data, and the *χ*^2^ test for categorical variables. The discriminative capacity of the various cognitive approaches was assessed using a Receiver Operating Characteristic (ROC) curve analysis to determine the sensitivity, specificity and Area under the Curve (AUC) of the different cut-off scores. Sensitivity to change over time was addressed following several approaches as a function of the interest of the analysis. First, we addressed the mean change from baseline at each time-point and group for each cognitive instrument. The percentage of change from baseline was subjected to repeated measures ANOVA to test for the existence of any kind of interaction in terms of change as a function of group category, time-point and instrument. To address the existence of differences in the dynamics on the rate of change between groups in all of these cognitive measures, we also performed between-groups ANOVA analysis comparing the scores obtained between visits. To compare differences between groups in the mean change between visits, we used a generalized linear mixed-effects model using age, education and UHDRS-TMS. This statistical model was used to mitigate the data missing from the follow-up visits. We also calculated the rate of change from one cognitive category to another along visits using the gold-standard approach. Using a sensitivity-based and specificity-based approach, we then estimated the minimally clinical relevant change associated with an increased risk of moving from one cognitive category to another at 12 months. Finally, we determined the Minimal Clinically Important Difference (MCID) at 6 months and at 12 months for each cognitive variable using an approach based on the determination of the minimum score 1.645 SD away from the mean ± SD of the difference (CI = 90%). All analysis was performed using IBM-SPSS software (version 26; SPSS. Inc. Armonk, NY) and significance was set at *p* < 0.05.

## Results

### Clinical and sociodemographic data

The sample consisted of 180 HD gene-mutation carriers (mean age = 49.6 ± 11.7; mean years of education = 12.3 ± 4.4; mean TFC = 10.4 ± 2.8). As seen in Table 1, from the total HD sample, 33 participants were classified as PreHD (mean age = 40.9 ± 8.7; mean CAG = 42.7 ± 2.5; mean years of education = 14.5 ± 4.4; mean TFC = 12.9 ± 0.4), and 147 as manifest HD (mean age = 51.6 ± 11.4; mean CAG = 43.5 ± 2.9; mean years of education = 11.8 ± 4.2; mean TFC = 9.8 ± 2.8. No discrepancies were found between raters regarding the cognitive status of patients. According to the gold-standard, among the symptomatic HD group, 52 were classified as HD-NC (mean age = 49.5 ± 9.6; mean CAG = 42.8 ± 2.4; mean years of education = 14.4 ± 3.3; mean TFC = 12.1 ± 1.0), 49 were classified as HD-MCI (mean age = 52.3 ± 11.1; mean CAG = 43.4 ± 2.3; mean years of education = 11.2 ± 4.0; mean TFC = 9.9 ± 1.6) and 46 were classified as HD-Dem (mean age = 53.2 ± 13.4; mean CAG = 44.3 ± 3.7; mean years of education = 9.5 ± 3.8; mean TFC = 7.1 ± 2.7). No participants in the PreHD group were classified as having mild or severe cognitive impairment.

**Table 1:** Clinical and sociodemographic characteristics of the sample

	PreHD (*n* = 33)	HD-NC (*n* = 52)	HD-MCI (*n* = 49)	HD-Dem (*n* = 46)	*P*
Age	40.9 ± 8.7	49.5 ± 9.6	52.3 ± 11.1	53.2 ± 13.4	^a^0.003; ^b^< 0.001; ^c^< 0.001: ^d^0.585; ^e^0.340; ^f^< 0.974
Gender (female/male)	25/8	33/19	23/26	23/23	^a^χ = ^a^0.235; ^b^0.009; ^c^0.021: ^d^0.095; ^e^0.179; ^f^< 0.765
Education	14.5 ± 4.4	14.4 ± 3.3	11.2 ± 4.0	9.5 ± 3.8	^a^0.999; ^b^0.001; ^c^< 0.001; ^d^< 0.001; ^e^< 0.001; ^f^< 0.126
CAG	42.7 ± 2.5	42.8 ± 2.4	43.4 ± 2.3	44.3 ± 3.7	^a^0.993; ^b^0.702; ^c^0.069: ^d^0.782; ^e^0.059; ^f^< 0.388
DBS^1^	288.4 ± 98.5	349.1 ± 81.9	394.3 ± 97.9	430.6 ± 113.9	^a^0.036; ^b^< 0.001; ^c^< 0.001: ^d^0.102; ^e^< 0.001; ^f^0.281
UHDRS-TMS^2^	1.1 ± 1.6	16.8 ± 11.2	35.5 ± 15.0	47.9 ± 19.7	^a^< 0.001; ^b^< 0.001; ^c^< 0.001: ^d^< 0.001; ^e^ < 0.001; ^f^ < 0.001
TFC^3^	12.9 ± 0.4	12.1 ± 1.0	9.9 ± 1.6	7.1 ± 2.7	^a^0.162; ^b^< 0.001; ^c^< 0.001: ^d^< 0.001; ^e^< 0.001; ^f^< 0.001
Disease stage					
Premanifest	33	–	–	–	
Stage I	–	49	21	2	
Stage II	–	3	28	34	
Stage III	–	–	–	10	

^1^Disease burden score

^2^Unified Huntington’s disease rating scale-Total motor score

^3^Total functional capacity

^4^Mini-mental state examination

^5^Parkinson’s Disease-Cognitive Rating Scale

^a^PreHD vs HD-NC

^b^PreHD vs HD-MCI

^c^PreHD vs HD-Dem

^d^HD-NC vs HD-MCI

^e^HD-NC vs HD-Dem

^f^HD-MCI vs HD-Dem

### Discriminative capacity of the cognitive approaches

In terms of discriminating between cognitive categories, the validity and reliability of the PD-CRS, the MMSE and the UHDRS cogscore were determined based on Analysis Under The Curve (AUC). This analysis was restricted to the sample of symptomatic participants because all preHD participants were classified as cognitively intact.

**Fig. 1:**
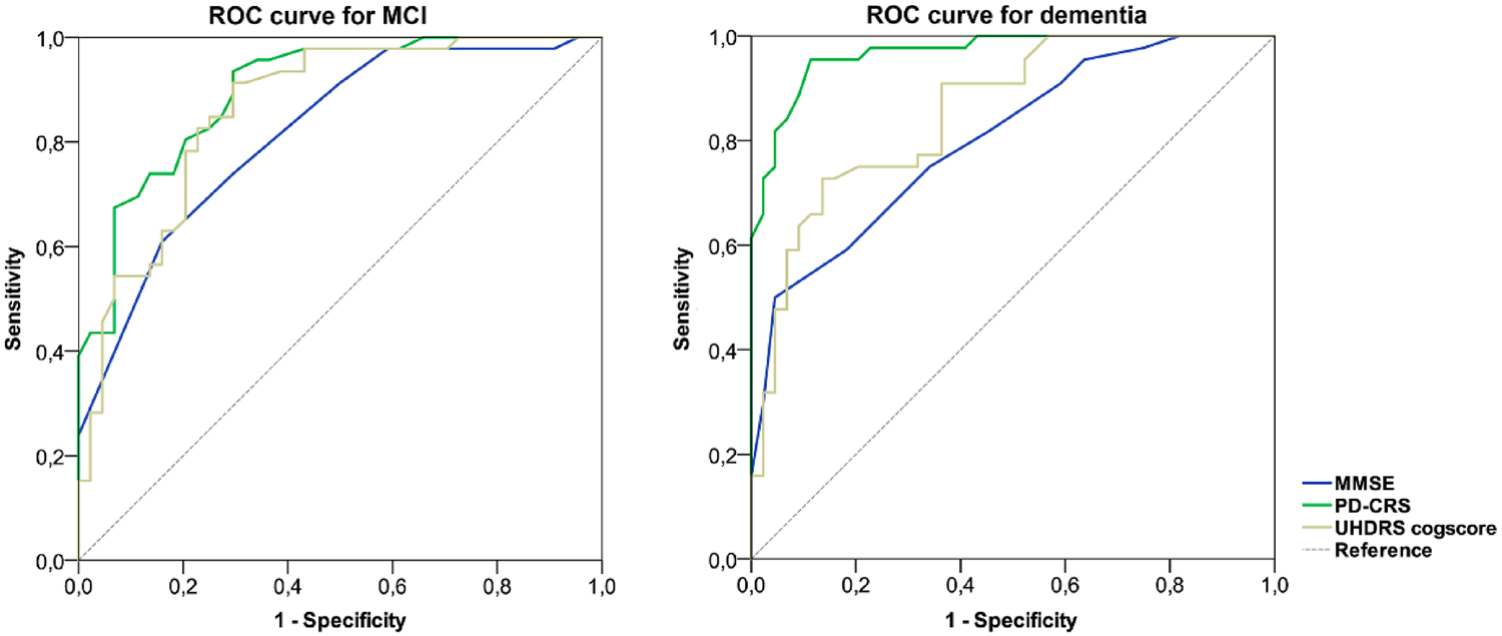
Receiver operating characteristic (ROC) curves illustrating the discriminative properties of the PD-CRS, the MMSE and the UHDRS cogscore

As seen in Fig. 1, a PD-CRS total score of 81/82 had a sensitivity of 0.83 and specificity of 0.81 (AUC = 0.898) to discriminate HD patients in the NC group from those in the MCI group. A MMSE total score of 25/26 had a sensitivity of 0.97 and a specificity of 0.39 (AUC = 0.816). Regarding the UHDRS cogscore, a score of 165/166 achieved a sensitivity of 0.84 and a specificity of 0.75 (AUC = 0.863). Focusing on the category of dementia, we found a PD-CRS total score of 61/62 had a sensitivity of 0.88 and specificity of 0.93 (AUC = 0.968). The MMSE total score 20/21 had a sensitivity of 0.97 and specificity of 0.36 (AUC = 0.803), and a UHDRS cogscore of 93/95 had a sensitivity of 0.90 and specificity of 0.54 (AUC = 0.856) to discriminate between the categories MCI and dementia.

Bivariate correlation analysis showed the expected significant associations between the total scores of the various cognitive instruments, with the strongest associations being found between the PD-CRS total score and the UHDRS cogscore (*r* = 0.886; *p* < 0.0001), and between the PD-CRS total score and the MMSE (*r* = 0.742; *p* < 0.0001). The MMSE showed a mild but significant association with the UHDRS cogscore (*r* = 0.656; *p* < 0.0001).

### Sensitivity to change over time

Sensitivity to change was addressed in the available population at 6 and 12 months.

Table 2 depicts the mean change at each time-point for each of the cognitive measures in each group based on a linear mixed-effects model using age, education and UHDRS-TMS as covariables. Repeated measures ANOVA showed a significant time × group interaction in terms of rate of change for the PD-CRS [*F*(6,59) = 2.337; *p* = 0.039], but not for the MMSE or for the UHDRS cogscore.

**Table 2:** Linear mixed-effects models with covariables (age, education and UHDRS)

	PreHD					HD-NC				
	Baseline	6 months	12 months	*P* global	*P* dif groups	Baseline	6 months	12 months	*P* global	*P* dif groups
PD-CRS^5^ total score	105.7 ± 1.9	107.7 ± 1.9	106.6 ± 2.2	0.211	0.438/1.000/1.000	93.2 ± 1.6	95.6 ± 2.2	92.6 ± 2.2	0.101	0.090/0.654/0.043
MMSE^4^	28.8 ± 0.3	29.4 ± 0.4	28.9 ± 0.4	0.204	0.249/1.000/0.953	28.4 ± 0.2	28.5 ± 0.4	28.5 ± 0.3	0.944	0.842/0.753/0.965
UHDRS Cogscore	262.3 ± 6.5	269.5 ± 7.7	262.1 ± 7.1	0.443	1.000/1.000/0.657	211.2 ± 7.0	216 ± 7.5	206 ± 7.9	0.286	0.984/0.140/0.215

	HD-MCI					HD-DEM				
	Baseline	6 months	12 months	*P* global	*P* dif groups	Baseline	6 months	12 months	*P* global	*P* dif groups
PD-CRS^5^ total score	72.9 ± 1.5	72.3 ± 2.2	67.0 ± 2.1	0.003	0.679/0.001/0.019	46.8 ± 1.6	45.4 ± 1.8	41.8 ± 1.7	0.003	0.387/0.001/0.021
MMSE^4^	25.9 ± 0.3	24.3 ± 1.4	25.3 ± 0.4	0.189	0.202/0.085/0.369	22.3 ± 0.5	22.7 ± 0.7	21.0 ± 0.6	0.042	0.390/0.027/0.014
UHDRS Cogscore	142.6 ± 6.1	140.6 ± 7.8	133.1 ± 7.2	0.076	0.715/0.023/0.203	88.6 ± 4.6	81.1 ± 5.9	77.6 ± 6.8	0.231	0.180/0.103/0.538

For the PD-CRS, in the preHD group, there was a significant change between baseline and 6-month visit [*t*(33) = − 2.2; *p* = 0.045] in terms of an improvement of 2.9 ± 5.3 points, mostly attributed to learning effects. No significant differences were found between other visits, either for the PD-CRS or the other instruments.

Focusing on the HD-NC, we observed a significant change between 6 and 12 months [*t*(52) = 2.5; *p* = 0.028] in terms of an improvement of 10.1 ± 16 points for the UHDRS cogscore. No significant differences were found between other visits with any instrument.

In the HD-MCI, there was a significant change for the PD-CRS between baseline and 12-month visit [*t*(49) = − 3.6; *p* = 0.001] in terms of a decrease of − 5.8 ± 8.4 points. We also noted a significant change between the 6-month and 12-month visit [*t*(49) = 2.2; *p* = 0.046] in terms of an improvement of 5.5 ± 9.7 points. No significant differences were found between baseline and 6-month visit. For the MMSE, no significant differences were found between visits. And for the UHDRS cogscore, significant differences were found between baseline and 12-months [*t*(49) = 2.2; *p* = 0.035] in terms of an improvement of 9.0 ± 19.3 points. No significant differences were found between baseline and the 6-month visit, or between the 6-month and 12-month visit.

The HD-Dem showed a significant change for the PD-CRS between baseline and 12-month visit [*t*(46) = − 3.4; *p* = 0.002], consisting of a decrease of − 4.6 ± 7.7 points. No significant differences were found between other visits. For the MMSE and the UHDRS cogscore, no significant differences were found between any visits.

**Fig. 2:**
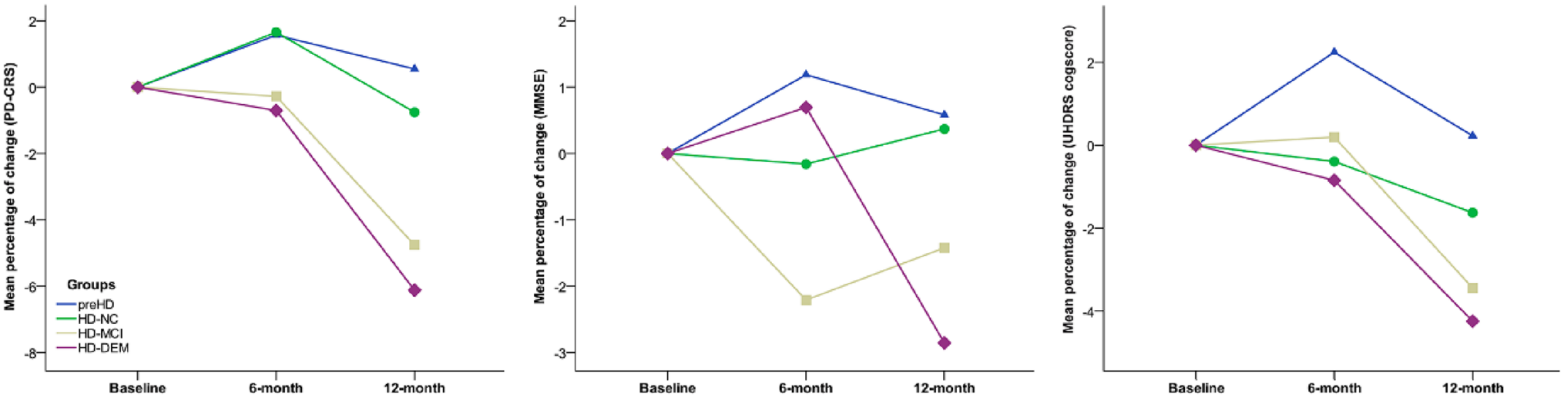
Cognitive trajectory in terms of percentage of change with respect to baseline along follow-up visits for each cognitive instrument and group

Figure 2 depicts the trajectories of changes (represented as percentage of change) in cognitive measures and groups. Both the HD-MCI and HD-Dem groups showed a pattern of progressive worsening, clearly captured with the PD-CRS and with the UHDRS cogscore, but not with the MMSE. In the preHD group, no instrument captured significant decreases in performance, but learning effects were observed between baseline and 6-month follow-up.

At the 12-month visit we calculated the rate of conversion from one category to another, both for the HD-NC and HD-MCI groups. For the HD-NC group, 7.7% of participants converted to HD-MCI, and 1.9% converted to HD-Dem. In the HD-MCI group, the proportion of participants who converted to HD-Dem reached 16.3%. To estimate the minimally clinical relevant change associated with an increased risk for moving from one category to another at 12 months, we performed a sensitivity-based and specificity-based approach. In the overall sample of symptomatic participants, ROC curves indicated that a change of 9/10 points in the PD-CRS total score indicated a significant change associated with moving to another cognitive category (sensitivity = 0.984; specificity = 0.800, AUC = 0.972; 95% CI 0.935–1.000). Regarding the other cognitive measures, we found an AUC = 0.436 for the MMSE, and AUC = 0.323 for the UHDRS cogscore. Thus, these measures did not reach minimal significance of AUC > 0.5.

Regarding each category, in the HD-NC group, a change of 5/6 points in the PD-CRS (sensitivity = 0.875; specificity = 1, AUC = 0.981; 95% CI 0.939–1.000) was associated with the transition to another cognitive category. With an AUC = 0.419 and an AUC = 0.452, neither the MMSE nor the UHDRS cogscore reached minimal significance. In the HD-MCI, a decrease of 7/8 points in the PD-CRS (sensitivity, 0.850%; specificity 1.000%, AUC = 0.975; 95% CI 0.927–1.000) indicated a significant change. The AUC for the other cognitive measures did not reach significance (AUC = 0.412 for the MMSE, and AUC = 0.176 for the UHDRS cogscore).

Regarding the MCID, Table 3 shows the minimum change with a 90% CI for being a significant change between visits, for each cognitive instrument and group.

**Table 3:** Minimally Clinically Important Difference (MCID) for each group, timepoint, and instrument

	preHD		Overall sample (symptomatic)		HD-NC		HD-MCI		HD-Dem	
	6-month	12-month	6-month	12-month	6-month	12-month	6-month	12-month	6-month	12-month
PD-CRS	9.8	10.7	9.9	13.4	12.6	10.8	9.4	15.8	10.5	13.8
MMSE	3.7	3.4	5.9	4.3	3.7	3	8.9	4	3.9	5.9
Cogscore	45.2	20.8	22.8	29.3	19.3	24	23.3	29.8	28.6	40.1

## Discussion

In this study, we aimed to determine the discriminative properties of cognitive instruments to establish a more reliable approach to the study of cognitive status in HD patients. We addressed the sensitivity of these instruments to change at different time-points, determined the rate of conversion among categories, and computed the minimum change expected to occur in the different scales as a function of an increased risk for worsening in terms of category conversion.

Our results favor the PD-CRS over the MMSE and the UHDRS cogscore, and provide reliable cutoff scores to differentiate cognitive categories in HD. Notably, these cutoff scores were equivalent to those obtained in a previous study and cohort, and showed excellent sensitivity and specificity values. Our results confirm both the validity of the PD-CRS to assess global cognitive status in HD individuals and the reliability of cutoff scores of 81/82 to discriminate HD subjects with MCI and with dementia, respectively. For the MMSE, the common cutoffs of 25/26 and 20/21, respectively, showed the best discriminative capacity between MCI and dementia. However, the sensitivity and specificity values associated with these cutoffs were significantly lower than the minimum standards required to consider the instrument valid [34]. Thus, according to our results, the MMSE does not appear to qualify as a suitable instrument to detect MCI and/or dementia in the context of HD.

The UHDRS cogscore has been used for several years in the context of multicenter observational clinical trials [18, 35]. Several measures comprising this composite score are extremely sensitive to overall disease progression as has been shown in previous studies. However, to date, no study has provided a meaningful clinical significance of the UHDRS cogscore in terms of its use to determine the cognitive status of the patients. Our study fills this gap and shows that the UHDRS cogscore cutoffs obtained for MCI and dementia have an acceptable sensitivity and specificity relationship. However, the UHDRS cogscore is still a combination of tests based primarily on executive, attentional and psychomotor processes that certainly do not cover the broad spectrum of cognitive impairment that can be heterogeneously present in HD people. Therefore, although our findings indicate that it is useful to discriminate between cognitive categories, a more global scale should be preferred when the objective is also to explore the global cognitive status of an HD subject.

In relation to sensitivity to change, it is noteworthy that the only instrument that showed significant differences in the rate of progression at 6 and 12 months was the PD-CRS. This means that while the UHDRS cogscore or any measure sensitive to disease progression changes over time, the trajectory of this change when using instruments such as the MMSE or the UHDRS cogscore is the same regardless of the cognitive status of the patients. Because it is conceptually evident that the trajectory of cognitive changes can be expected to differ depending on the cognitive status of the patients, it could be assumed that, from the point of view of cognitive assessment, an efficient instrument should be able to distinguish trajectories of the different cognitive groups. This does not occur in the case of the MMSE or the UHDRS cogscore, but it is evident and significant in the case of the PD-CRS. Specifically, the PD-CRS is the only instrument that showed significant involutional changes throughout the 12-month follow-up in both the HD-MCI group and the HD-Dem group.

Regarding the rate of conversion from one cognitive category to another one, we provide evidence that in our cohort, during the follow-up period, 7.7% of participants from the HD-NC group converted to HD-MCI, 1.9% converted to dementia, and 16.3% converted from HD-MCI to dementia. We consider that taking these category changes into account is relevant once we have been able to show that the trajectory of cognitive measures differs depending on the category to which the subjects belong. This implies that when monitoring the trajectory of HD patients (i.e., in the context of a clinical trial) it is essential to know each patient’s cognitive status to be able to accurately understand the meaning of the changes or stability observed in the measures used.

Analysis of the changes in the various cognitive measures associated with the rate of conversion from one category to another showed that a change of 9/10 points in the PD-CRS was associated with a change in cognitive category regardless of the category to which the subjects belonged. Focusing on individuals in the HD-NC group, a 5/6point change was reliably associated with conversion from one category to the other, whereas in the HD-MCI group, a 7/8-point change was required for transition to dementia. These measures of relevant cognitive changes were statistically valid only for the PD-CRS, and the values obtained for the MMSE or the UHDRS cogscore did not reach the minimum required. In this regard, this does not call into question the demonstrated sensitivity of the UHDRS cogscore to track disease progression, but it does demonstrate that it lacks validity for the purpose of tracking conversion to MCI or to dementia.

Finally, we considered it useful to study the Minimal Clinically Important Difference (MCID) for each instrument in the whole sample and in each group. This approach allows us to determine whether an observed change—regardless of whether we are talking about transition or change of cognitive category—is statistically significant. This is particularly useful in the context of monitoring disease progression, such as in interventional studies. Our results show that the MCID is different for each instrument, group, and time point. Therefore, when these instruments are used in the monitoring of cognitive status in people with HD, they must be adjusted according to the time interval studied and the group or cognitive category.

In summary, current diagnostic criteria for the cognitive syndromes in HD are limited and the need for reliable instruments to assess cognitive status is evident. The PD-CRS shows excellent discriminative properties that have been replicated in two studies and in two different cohorts [4, 23]. Despite the lack of specific definitions of MCI and dementia in HD, this tool shows adequate discrimination between cognitive groups. It is sensitive to change both in relation to conversion from one cognitive category to another and also in relation to monitoring the progression of cognitive deterioration. Besides, it is the only instrument available to date that is able to show the different trajectories of cognitive deterioration. In parallel, we provide the cutoffs that can be used for the PD-CRS, MMSE, and UHDRS cogscore to discriminate between cognitive groups. Especially with regard to the UHDRS cogscore, we believe our findings are particularly useful as these tests continue to be widely used in observational studies of large cohorts of patients (i.e.: Enroll-HD). Having this approximation based on the cutoffs thus allows us to exploit the data from this study according to this type of classification.

We are fully aware that the PD-CRS is merely a screening approach for overall cognitive status, which, although it has several strengths compared to other screening instruments, is not without limitations. Cognitive manifestations in HD encompass changes in processes that are not covered by the PD-CRS. This means that a comprehensive assessment of patients’ cognitive status should follow the standards of neuropsychological assessment, taking into account the role of processes such as certain language impairments [36, 37] or social cognition [38–40], as well as specific neuropsychiatric manifestations [41, 42]. Also, the PD-CRS does not explore relevant processes in the HD context such as psychomotor speed and word reading [11, 43]. However, further analysis of present data showing that a global scale such as the PD-CRS performs well in an HD population may serve to complement or recombine current instruments and contribute to the design of new tools.

This study has other points to consider. First, other instruments, such as the MoCA, could be explored in future studies for their discriminative capacity and sensitivity to change in HD. Second, the restrictions imposed by the pandemic contributed to an unexpectedly high rate of follow-up losses that may have somehow influenced some results. Nevertheless, a considerably representative sample was still examined at the three time points of the study.
